# Unconventional posttranslational modification in innate immunity

**DOI:** 10.1007/s00018-024-05319-8

**Published:** 2024-07-06

**Authors:** Jiaxi Chen, Dejun Qi, Haorui Hu, Xiaojian Wang, Wenlong Lin

**Affiliations:** 1 The Second Affiliated Hospital and Institute of Immunology, Zhejiang University School of Medicine, Hangzhou, 310058 Zhejiang China; 2 Institute of Immunology and Bone Marrow Transplantation Center, The First Affiliated Hospital, School of Medicine, Zhejiang University, Hangzhou, 310003 China

**Keywords:** Unconventional posttranslational modifications, Innate immunity

## Abstract

Pattern recognition receptors (PRRs) play a crucial role in innate immunity, and a complex network tightly controls their signaling cascades to maintain immune homeostasis. Within the modification network, posttranslational modifications (PTMs) are at the core of signaling cascades. Conventional PTMs, which include phosphorylation and ubiquitination, have been extensively studied. The regulatory role of unconventional PTMs, involving unanchored ubiquitination, ISGylation, SUMOylation, NEDDylation, methylation, acetylation, palmitoylation, glycosylation, and myristylation, in the modulation of innate immune signaling pathways has been increasingly investigated. This comprehensive review delves into the emerging field of unconventional PTMs and highlights their pivotal role in innate immunity.

## Introduction

Innate immunity serves as the first line of defence against pathogen infection by discriminating between various pathogen-associated molecular patterns (PAMPs) and danger-associated molecular patterns (DAMPs) [1]. PAMPs include microbial products, such as lipopolysaccharides and glucans/chitin, whereas DAMPs contain endogenous stress signals, such as uric acid and extracellular ATP. Pattern recognition receptors (PRRs) are critical for tailoring immune responses to PAMPs and DAMPs. Based on their localization and function, PRRs are classified as membrane-bound PRRs, including Toll-like receptors (TLRs) and C-type lectin receptors (CLRs), as well as cytoplasmic PRRs, comprising NOD-like receptors (NLRs) and retinoic acid-inducible gene I (RIG-I)-like receptors (RLRs) [2]. In addition, formyl peptide receptors, complement receptors, signaling lymphocyte activation molecules (SLAMs), and numerous nucleic acid sensors have been demonstrated to be key PRRs for maintaining immune homeostasis.

**Fig. 1:**
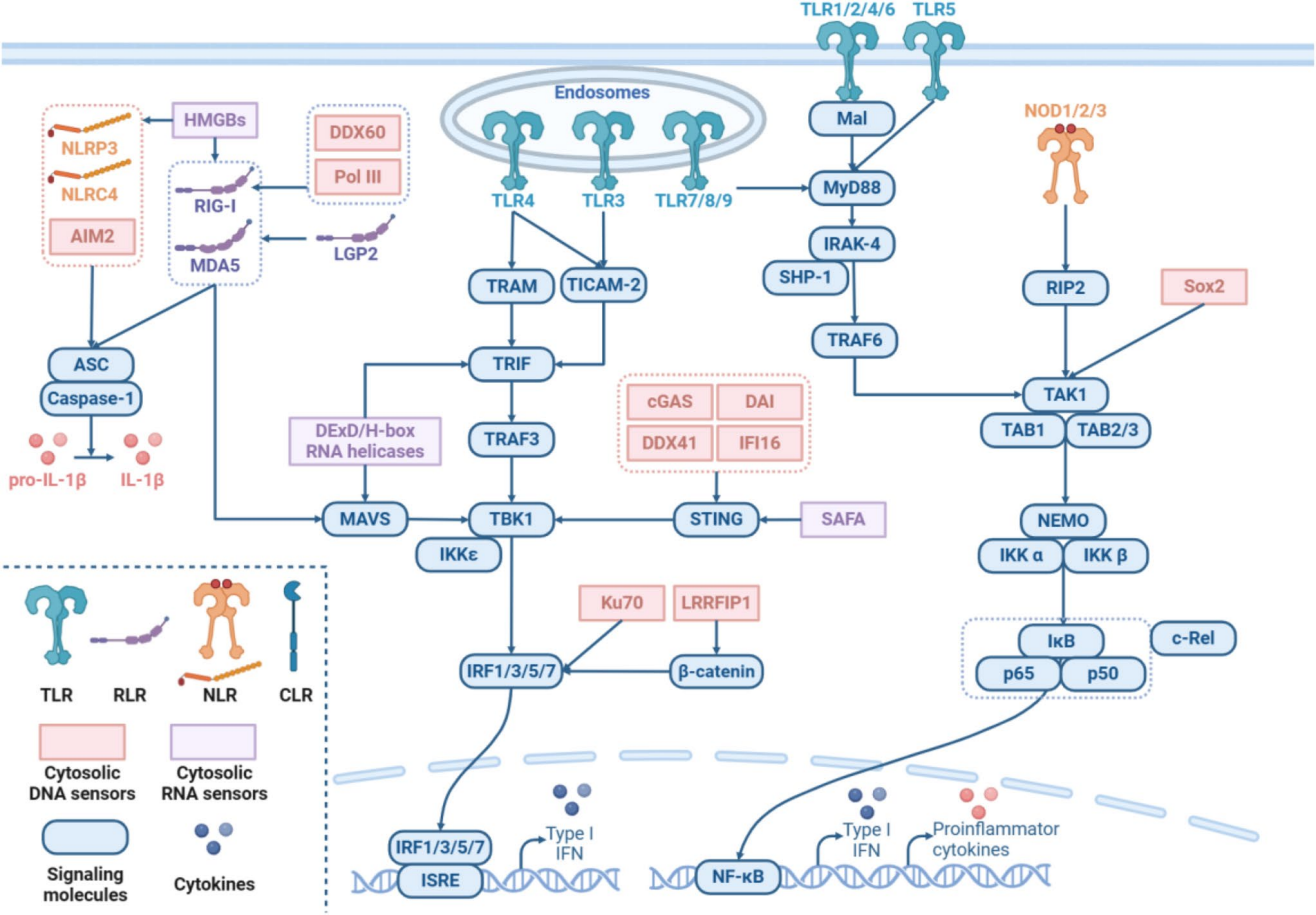
Schematic representation of pattern-recognition receptor (PRR) signaling pathways. A complex signaling network is triggered by multifarious PRRs, including Toll-like receptors (TLRs), nucleotide oligomerization domain (NOD)-like receptors (NLRs), retinoic acid-inducible gene-I (RIG-I)-like receptors (RLRs), C-type lectin receptors (CLRs), and numerous other nucleic acid sensors, which are presented through icons of different shapes and colors. The blue boxes represent key molecules in PRR signaling pathways, and the line with an arrow indicates a cascade of responses. The double horizontal lines at the top represent cell membranes, while the curved dotted line at the bottom represents the nuclear membrane

In this regard, TLRs are the best-studied PRRs that recognize bacterial lipopolysaccharides and viral double-stranded RNA. Ten members of the TLR family, which transduce signals in myeloid differentiation primary response gene 88 (MyD88)-dependent or MyD88-independent pathways, have been identified in humans [3]. The former starts with MyD88, a TIR domain-containing adaptor, leading to the recruitment of IL-1 receptor-associated kinase-4 (IRAK-4) and then TNF receptor-associated factor 6 (TRAF6), resulting in the activation of the IKK complex and NF-κB. The latter occurs via TIR domain-containing adaptor molecule 1 (TRIF) and TNF receptor-associated factor 3 (TRAF3), which activate IKKε/TANK-binding kinase 1 (TBK1), contributing to the phosphorylation of interferon regulatory factor 3 (IRF3)/IRF7 and subsequent expression of interferon-β (IFN-β). RLRs, including retinoic acid-inducible gene I (RIG-I), melanoma differentiation-associated gene 5 (MDA5), and the laboratory of genetics and physiology 2 (LGP2), are also essential for the detection of viral RNA [4]. RIG-I and MDA5 activate IPS-1/MAVS on mitochondria by interacting with TBK1, further promoting IRF3- or IRF7-dependent expression of IFNs. In addition, diverse cytoplasmic DNA sensors activate IRF3 through the stimulator of interferon response cGAMP interactor 1 (STING)-TBK1 axis, such as cyclic GMP-AMP synthase (cGAS), DEAD (AspGlu-Ala-Asp) box helicase 41 (DDX41), IFN-inducible protein-16 (IFI16), and DNA-dependent activator of IRFs (DAI) [5]. Intracellular DNA also triggers a protease cascade through the assembly of the AIM2 inflammasome and caspase-1-mediated cleavage of members of the IL-1β family [6]. The NLR family comprises 22 human proteins that are equipped with C-terminal leucine-rich repeats (LRRs), such as nucleotide-binding oligomerization domain-containing protein 1 (NOD1), NOD2, LRR, PYD domain-containing protein 3 (NLRP3), and NLR family CARD domain-containing protein 4 (NLRC4). NOD1 and NOD2 activate NF-κB via the receptor-interacting serine/threonine kinase 2 (RIP2)-TGF-β activated kinase 1 (TAK1)-IKK axis, triggering inflammasome activation. Other cytoplasmic RNA sensors include DExD/H-box RNA helicases, HMGBs and SAFAs [7]. These sensors mediate IFN induction mainly through MAVS but also through RIG-I, STING, and TRIF [8] (Fig. 1).

Sophisticated mechanisms have been developed to precisely modulate the activation and amplitude of PRR signaling, among which posttranslational modifications (PTMs) have attracted increasing attention. Widely involved in maintaining immune homeostasis, PTMs regulate the structure, stability, activity, localization, and interaction of innate immune sensors and key signaling proteins with other biomolecules. Dysregulation of PTMs has been implicated in the pathogenesis of several disorders, including diseases. PTMs consist of conventional PTMs, such as phosphorylation and ubiquitination and unconventional PTMs, such as unanchored ubiquitination, ISGylation, SUMOylation, NEDDylation, methylation, acetylation, palmitoylation, glycosylation, and myristylation [9]. In this review, we provide an overview of the functions underlying the different types of unconventional PTMs that occur in the context of innate immunity.

### Unanchored ubiquitination in innate immunity

Unanchored ubiquitination mediated by unanchored ubiquitin (Ub) is a new unconventional form of PTM that is poorly understood but has been shown to have a critical physiological function in regulating immune signaling pathways. In contrast to well-characterized covalently linked ubiquitination, the unanchored variant is unusual in that it is not directly bound to a substrate and can function as a three-dimensional PTM signal that is amplified by multiple noncovalent interactions. These unanchored ubiquitin chains have been shown to play a critical role in the regulation of immune signaling pathways, particularly in antiviral innate immunity. They act as dynamic and spatially flexible posttranslational modification (PTM) signals that can be recognized and interpreted by multiple proteins involved in immune signaling.

Like covalently linked ubiquitination, unanchored ubiquitination includes K63-linked and K48-linked ubiquitination. TLRs and RLRs are the major targets of unanchored ubiquitin chains. Unanchored K63-linked polyubiquitination occurs in the context of both TLR and RLR signaling. Upon IL-1β stimulation, the ubiquitin ligase TRAF6 catalyzes K63-linked polyubiquitination with a ubiquitin-binding enzyme complex consisting of UBC13 (known as UBE2N), UEV1A (UBE2V1) and UBCH5C (known as UBE2D3), which results in an unanchored K63-linked polyubiquitin chain that directly activates TAK1 (also known as MAP3K7) and downstream IκB kinase (IKK) and NF-κB essential molecule (NEMO) and ultimately activates NF-κB by binding to the ubiquitin receptor TAB2 (also known as MAP3K7IP2) [10]. However, unanchored K63-linked polyubiquitin chains can also efficiently modulate RLR signaling. During viral infection, the retroviral capsid lattice, including the HIV-1 capsid lattice, can be sensed by TRIM5. The retroviral capsid lattice enhances the UBC13-UEV1A-dependent E3 activity of TRIM5 and promotes TRIM5 to catalyze the synthesis of unbound K63-linked ubiquitin chains, which activate the TAK1 kinase complex and stimulate AP-1 and NF-κB signaling [11]. RNA containing 5’-triphosphate activates the RIG-I-IRF3 signaling cascade in a reconstituted system composed of RIG-I, mitochondria and the cytosol. RIG-I binds specifically to K63-linked polyubiquitin chains through its tandem CARD domains in an RNA- and ATP-dependent manner [12]. In addition, Ube2N, the main ubiquitin-conjugating enzyme for MAVS, cooperates with the E3 ligases Riplet and TRIM31 to promote unanchored K63-linked polyubiquitination of MAVS. K63-linked polyubiquitin chains loaded on MAVS can be directly recognized by RIG-I to initiate RIG-I-mediated MAVS aggregation. However, USP10, a direct deubiquitinating enzyme, removes unattached K63-linked polyubiquitin chains from MAVS, thereby attenuating RIG-I-mediated MAVS aggregation and type I interferon production [13]. K63-linked polyubiquitin chains can also induce the formation of a RIG-I tetramer composed of four RIG-I domains and four ubiquitin chains, which can cause the aggregation of MAVS CARDs and promote the activation of IKK and TBK1, resulting in the enhanced production of type I interferon [14]. Similarly, unanchored K63-linked polyubiquitin chains can promote the formation of heterotetrameric MDA5-CARD complexes, enabling the transmission of MDA5-MAVS signaling, which is essential for antiviral responses and immune homeostasis [15]. As a polymerase cofactor, the C-terminal IID of VP35 interacts with unanchored K63-linked polyubiquitin chains to enhance VP35 function and promote Ebola virus polymerase activity and replication. Ectopically expressed isopeptidase T (USP5) degrades unanchored polyubiquitin chains, reducing the association of VP35 with ubiquitin chains [16].

**Fig. 2:**
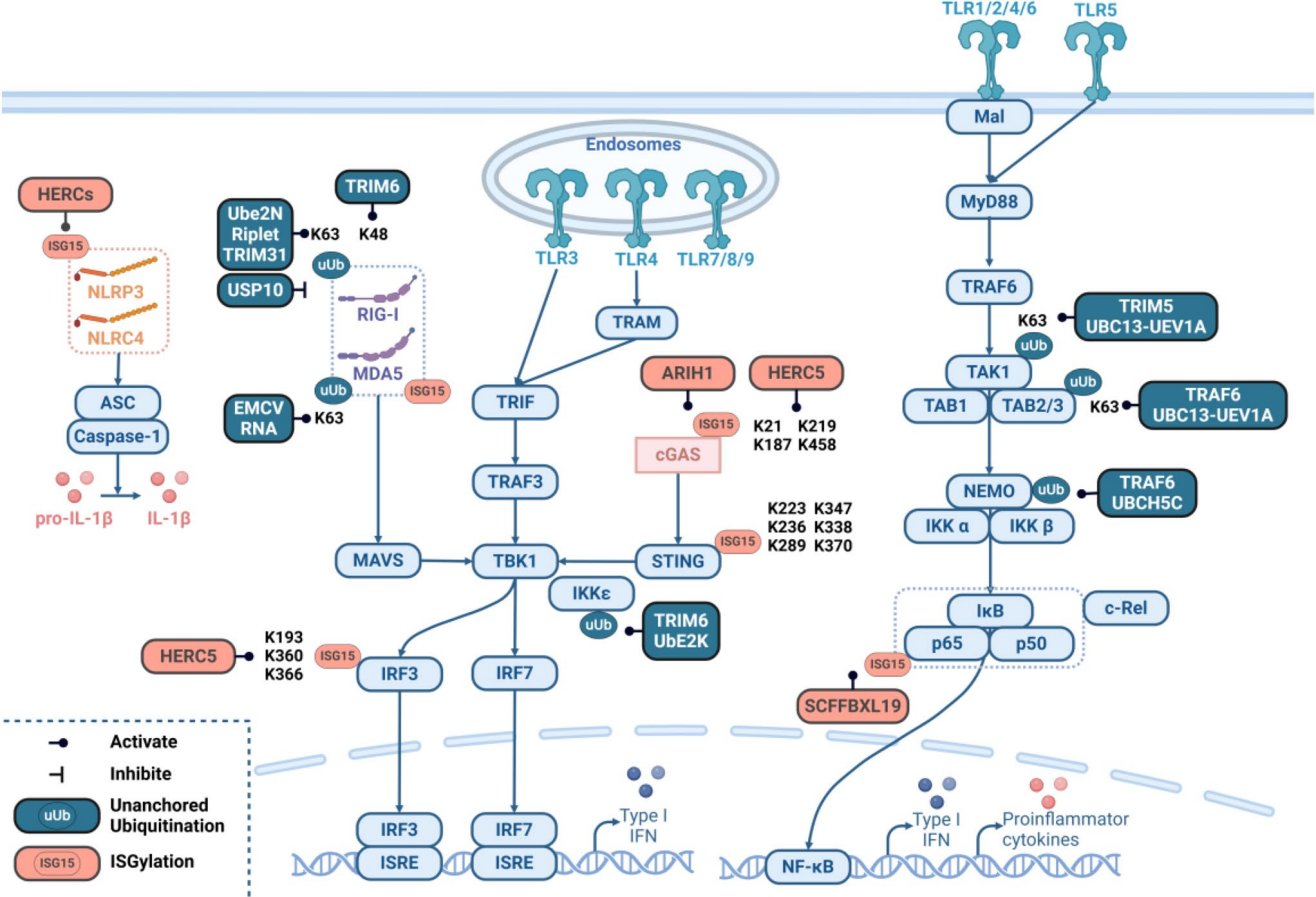
Regulation of PRR signaling pathways by unanchored ubiquitination and ISGylation. Unanchored ubiquitination and ISGylation occur through similar enzymatic cascades and often modify the lysine of target proteins. Dark cyan and pink indicate unanchored ubiquitination and ISGylation, respectively, of the proteins used on their targets. The blue boxes represent key molecules in PRR signaling pathways. A line with a dot indicates positive regulation, a line with a vertical bar indicates negative regulation, and the modification site is labeled next to it

In addition to unanchored K63-linked polyubiquitination on TLRs and RLRs, K48-linked polyubiquitination of other key proteins involved in TLR and RLR signaling has been demonstrated. TRIM6, a member of the E3 ubiquitin ligase tripartite motif (TRIM) protein family, cooperates with the E2 ubiquitin-coupled enzyme UBE2K to synthesize unanchored K48-linked polyubiquitin chains and activates IKKε and downstream induction of IKKε-dependent ISG and IKKε-dependent IFN induction, thereby establishing an effective antiviral state in cells [17]. The RNA helicase DHX16, a potential pattern recognition receptor (PRR), promotes IFN-I production via RIG-I and unanchored K48-linked polyubiquitin chains synthesized by the E3-Ub ligase TRIM6 [18] (Fig. 2). Whether the other types of unanchored polyubiquitination that correspond to conventional polyubiquitination, such as unanchored K6-, K11-, K27-, K29-, and K33-linked polyubiquitination, could be discovered in the future requires further advances in the field of biological technology, including the development of commercialized antibodies that specifically target these subtypes.

### ISGylation in innate immunity

ISGylation involves the attachment of ISG15, a ubiquitin-like protein (UBL) conserved among vertebrates, to lysine residues in substrate proteins. ISGylation occurs via a ubiquitin-like enzymatic cascade comprising E1, E2, and E3, distinct from the ubiquitylation that plays a critical role in innate immune responses.

Among all the ISGylation modifications on key proteins of the innate immune signaling pathway, ISGylation of the RLR signaling pathway has been extensively studied. Ariadne RBR E3 ubiquitin protein ligase 1 (ARIH1, also known as HHARI) promotes antiviral immunity and autoimmunity by inducing ISGylation and oligomerization of cGAS at its K187 residue [19]. HECT domain- and RCC1-like domain-containing protein 5 (HERC5) catalyzes ISGylation of IRF3 (at residues K193, K360, and K366) and cGAS (at residues K21, K187, K219, and K458), resulting in sustained IRF3 and cGAS activation [20, 21]. ISG15 modification also plays an important role in regulating STING activity via DNA recognition at residues K224, K236, K289, K347, K338, and K370, particularly residue K289, which is critical for STING activation and represents an important regulatory step in viral DNA recognition and autoimmune responses [22]. The RNA sensor MDA5 is ISGylated in its CARD domains, promoting MDA5 oligomerization and antiviral signaling [23, 24].

With respect to TLRs and NLRs, both CRISPR-based and pharmacologically mediated inhibition of IKKβ blocked the induction of ISG15 and BST2 [25]. Mechanistically, the SCF (Skp1-Cul1 F-box) protein E3 ligase SCFFBXL19 was identified as a novel ISG15 E3 ligase that targets and catalyzes ISGylation of the NF-κB p65 subunit, inhibiting its phosphorylation [26]. In addition, the predominant E3 ISGylation ligase in HERCs promoted NLRP3 ISGylation and inhibited K48-linked ubiquitination and proteasomal degradation, resulting in enhanced NLRP3 inflammasome activation [27] (Fig. 2).

### SUMOylation in innate immunity

SUMOylation is a process similar to ubiquitination that involves the transfer of small ubiquitin-like modifier (SUMO) molecules from the SUMO-specific enzymes E1, E2, and E3 to target substrate proteins. As a type of unconventional PTM, SUMOylation regulates protein localization and interactions with other binding partners, thereby affecting downstream signaling [28].

Previous evidence suggests that SUMOylation plays a critical role in regulating the innate immune response by targeting key signaling proteins of TLRs. The SUMO E3 ligases protein inhibitor of activated STAT (PIAS) and Ubc9 mediate the SUMOylation of TRAF6 and inhibit TRAF6 polyubiquitination-induced NF-κB activation in TLR signaling [29, 30]. SUMO-specific protease 6 (SENP1) induces the de-SUMOylation of the NEMO at K277/309, leading to attenuated NF-κB activation and downstream cytokine production in multitype cells and microglia [31–34]. As key proteins of TLR signaling, the SUMOylation of TRAF6 and NEMO tightly modulates the inflammatory response-dependent or independent polyubiquitination of the target substrate for the lysine modification site.

In addition to the SUMOylation of TLRs, many RLRs have been found to be targets of SUMOylation. During viral infection, the E3 SUMO ligase protein inhibitor of activated STAT 2β (PIAS2β) and TRIM38 positively regulate the SUMOylation of RIG-I and MDA5 at the K43/K865 and K96/K888 residues, respectively. SENP2 mediates de-SUMOylation at the same residues, ensuring proper protein levels of RIG-I and MDA5 to maintain innate immune signal transduction [35–37]. As a key adapter protein in RIG-I signaling, MAVS can be SUMOylated or de-SUMOylated at the K461 and K500 residues by PIAS3 or SENP1, respectively, promoting the aggregation of MAVS and the activation of IRF3 to prevent unnecessary auto- or extra-activation of innate antiviral immunity [38]. Nevertheless, the K694 residue of the crucial kinase TBK1 in RIG-I signaling can be de-SUMOylated by SENP1, thereby reducing antiviral activity [39]. PIAS1 promotes the SUMOylation of IRF3 and IRF7 at the K406 and K43 residues after Ebola virus infection, forming a part of the negative feedback loop of IFN signaling [40]. Moreover, SENP1 de-SUMOylates IRF8 by extracting SUMO3 from the K310 residue, converting IRF8 from a repressor to an activator in macrophages [41]. However, TRIM38 maintains the stability of the key DNA sensor cGAS and the adaptor protein STING during the early stages of DNA viral infection by catalyzing the SUMOylation of cGAS at residues K231, 470, 217, and 464 and of STING at K337, thereby promoting the cGAS/STING signaling-mediated innate immune response [42]. cGAS is de-SUMOylated by SENP2 in the late phase of infection or by SENP7 at residues K335, K372, and K382, facilitating its binding to DNA, oligomerization and nucleotide transfer, resulting in impaired expression of IFNs and ISGs in response to DNA activation [43, 44]. Taken together, the SUMOylation modification of RLR signaling reveals a complex mechanism for the multifunctional role of E3 enzymes, which can target different substrates; for example, SENP1 can de-SUMOylate MAVS, TBK1, and IRF8, thus affecting the antiviral immune response at different stages. Furthermore, many key proteins, such as cGAS and STING, can be modulated by different E3 enzymes to maintain the homeostasis of the antiviral response.

**Fig. 3:**
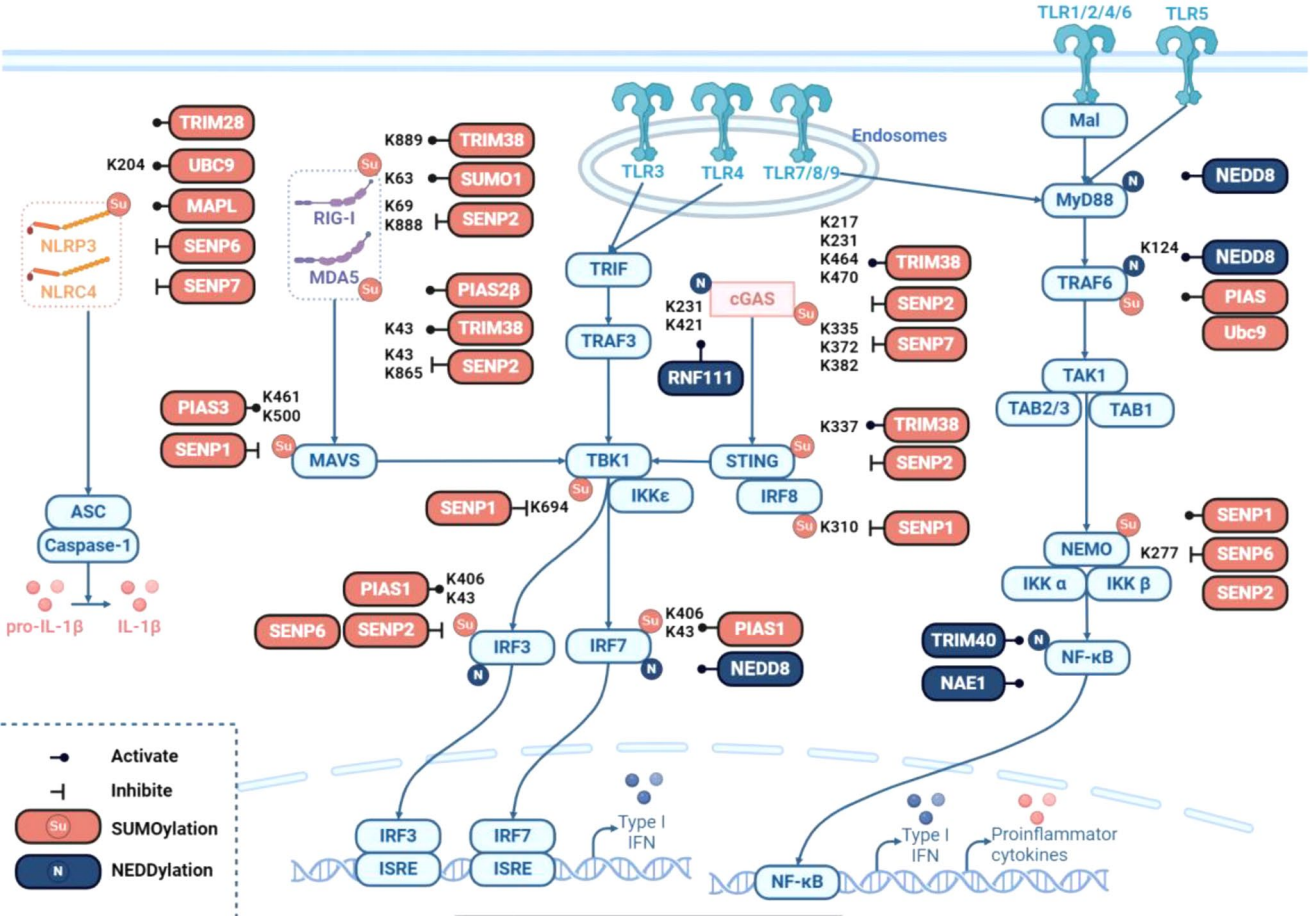
Regulation of PRR signaling pathways by SUMOylation and NEDDylation. SUMOylation and NEDDylation play a wide role in PRR signaling pathways, targeting receptors such as RIG-I, MDA5, NLRP3, and cGAS, as well as key molecules such as IRFs, NEMO, MAVS, and STING. Red and dark blue indicate SUMOylation and NEDDylation, respectively, of the proteins of interest. Light blue boxes represent key molecules in PRR signaling pathways. A line with a dot indicates positive regulation, and a line with a vertical bar indicates negative regulation. The modification site is labeled next to it. The amino acid residue involved in SUMOylation and NEDDylation is lysine (K)

With respect to NLRs, NLRP3 is SUMOylated by the SUMO E3 ligase MAPL and de-SUMOylated by the SUMO-specific proteases SENP6 and SENP7. De-SUMOylation of NLRP3 promotes NLRP3 activation without affecting NLRP3 protein stability [45]. However, TRIM28 SUMOylates NLRP3 to stabilize the NLRP3 protein and facilitate inflammasome activation [46] (Fig. 3), suggesting that SUMOylation of NLRP3 by different SUMO E3 ligases has opposite effects, suggesting that PTM-mediated therapies targeting the NLRP3 inflammasome should be more cautious.

### NEDDylation in innate immunity

NEDDylation, like ubiquitination, involves the attachment of the NEDD8 (neural precursor cell expressed developmentally downregulated protein 8) protein to specific lysine residues in target proteins. Like ubiquitination, NEDDylation regulates essential cellular processes by activating cullin-RING ligases (CRLs), a family of ubiquitin E3 ligases [47]. As NEDDylation regulates cellular processes, intricate modifications orchestrate immune responses against pathogens, highlighting the interconnectedness of basic biology and immune defence [48].

In the context of TLR signaling, the cullin E3 ubiquitin ligase SCF, the RING E3 ligase tripartite motif-containing protein 40 (TRIM40), and the sole regulatory subunit of the NEDD8 E1 enzyme NAE1, NEDDylate and regulate the ubiquitination-mediated degradation of the NF-κB precursor p105 subunit, NEMO, and NF-κB-inducing kinase (NIK), thereby negatively regulating NF-κB signaling [49–51]. NEDD8 negatively regulates MyD88 dimerization and suppresses MyD88-dependent NF-κB signaling by antagonizing its ubiquitination without affecting MyD88 protein stability [52]. However, in the RLR signaling pathway, NEDD8 mediates the NEDDylation of IRF3 and IRF7, contributing to the antiviral response in vitro and in vivo by promoting their transcriptional activity [53, 54]. MLN4924, a novel inhibitor of the NEDD8 activating enzyme, may prevent IRF3 binding to the IFN-β promoter, thereby inhibiting IFN-β production induced by IRF3 activation [55]. The NEDD8 E3 ligase RNF111 interacts with and poly-NEDDylates cGAS at the K231 and K421 residues, which in turn promotes RNF111 dimerization and enhances its DNA binding ability, ensuring proper activation of the cGAS-STING pathway [56] (Fig. 3). Thus, as lysine-targeted covalent PTMs, NEDDylation modifications on key proteins of TLRs and RLRs regulate signaling by either interfering with ubiquitination-mediated protein degradation or dimerization, suggesting the complex status of the target substrate in different cellular contexts.

### Methylation in innate immunity

Methylation includes both DNA methylation and protein methylation. As a posttranscriptional regulator of genes, DNA methylation has been extensively studied for its ability to control gene transcription. Moreover, protein methylation refers to the transfer of a methyl group from the donor s-adenosylmethionine (SAM) to the amino acid residues of the target proteins, with lysine (K) being the most commonly modified, as well as arginine (R) and cysteine (C). Protein methylation has many important biological functions, including gene regulation and signal transduction, and is, therefore, of increasing importance in the modulation of innate immune responses [57].

**Fig. 4:**
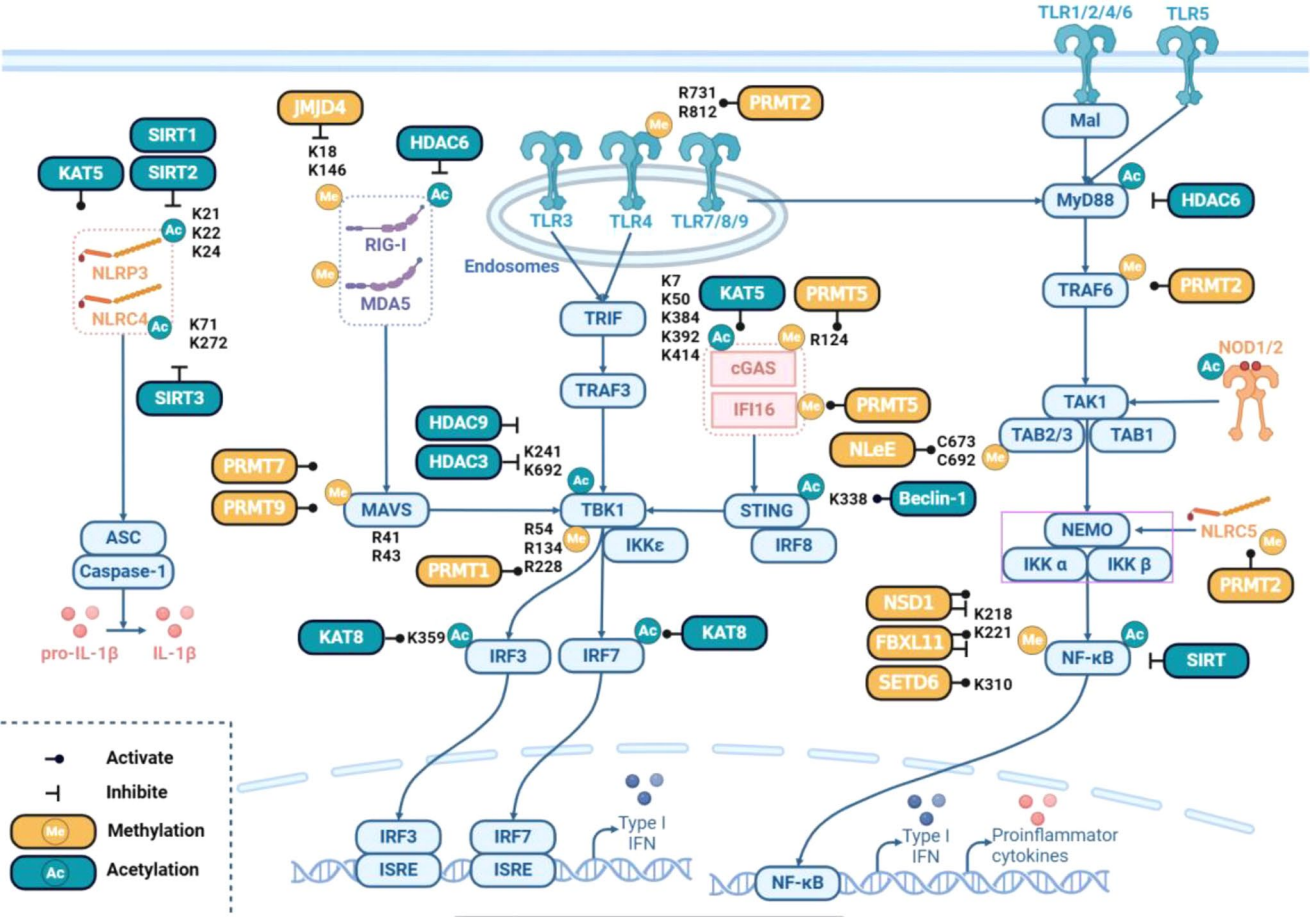
Regulation of PRR signaling pathways by methylation and acetylation. Methylation and acetylation occur widely in PRR signaling pathways. Yellow and cyan indicate the methylation and acetylation of the proteins used on their targets, respectively. The blue boxes represent key molecules in PRR signaling pathways. A line with a dot indicates positive regulation, and a line with a vertical bar indicates negative regulation. The modification site is labeled next to it, including the most modified lysine (K) and arginine (R) residues

Methylation and demethylation are important posttranslational modifications of the key signaling proteins of TLRs. Protein arginine methyltransferases (PRMTs) and methyltransferase SET domain-containing proteins, including SETDs, act as key modulators by methylating arginine and lysine residues in target proteins. Protein arginine methyltransferase 2 (PRMT2) methylates the TLR4 protein at the R731 and R812 residues, thereby enhancing the innate antiviral immune response [58]. In addition, PRMT2 hinders TRAF6 activation by stimulating arginine asymmetric dimethylation of TRAF6 at the R100 residue and subsequently preventing K63-linked TRAF6 autoubiquitination [59]. Methyltransferase SET domain-containing protein 6 (SETD6) regulates the monomethylation of the NF-κB p65 subunit at the K310 residue and inhibits the p65-induced inflammatory response [60]. Lysine methylase, nuclear receptor-binding SET domain-containing protein 1 (NSD1), and a lysine demethylase, F-box, and leucine-rich repeat protein 11 (FBXL11) control the activation of NF-κB by regulating lysine methylase and p65 demethylation at the K218 and K221 residues, respectively [61]. *Escherichia coli* NleE acts as a SAM-dependent methyltransferase that specifically modifies C673/692 in the TAB2/3-NZF domains, resulting in the loss of TAB2/3 ubiquitin binding and inhibition of host NF-κB signaling [62] (Fig. 4). Further research is needed to determine whether prokaryotic proteins such as *E. coli* NleE are widespread and can modulate PTMs in mammals to regulate the innate immune response.

In RLR signaling, RIG-I is constitutively monomethylated at K18 and K146. The demethylase JMJD4 mediates RIG-I demethylation and suppresses IL-6-STAT3 signaling [63]. Through direct interaction with TBK1, PRMT1 induces asymmetric methylation of TBK1 at the R54, R134, and R228 residues, increasing its oligomerization following viral infection and facilitating TBK1 phosphorylation, ultimately leading to increased production of type I interferons [64]. The role of arginine methyltransferase 5 (PRMT5) remains controversial. As a direct binding partner of cGAS, PRMT5 catalyzes the arginine symmetric dimethylation of cGAS at the R124 residue, and methylation of cGAS by PRMT5 attenuates the cGAS-mediated antiviral immune response by blocking the DNA binding ability of cGAS [65]. In the context of antitumor immunity, PRMT5-mediated methylation of IFN-γ inducible protein 16 (IFI16) or its murine homolog IFI204, which are components of the cGAS/STING pathway, attenuated cytosolic DNA-induced IFN and chemokine expression in melanoma cells. PRMT5 also inhibited the transcription of the gene encoding nucleotide-binding oligomerization domain-like receptor family caspase recruitment domain containing 5 (NLRC5), a protein that promotes the expression of genes involved in major histocompatibility complex class I (MHC I) antigen presentation [66]. Moreover, nucleus-localized cGAS interacts with PRMT5, which catalyzes the symmetric dimethylation of histone H3 arginine 2 at the *Ifnb* and *Ifna4* promoters, facilitating the access of IRF3 and the induction of type I interferons [67]. Nevertheless, PRMT5 inhibition impairs IFNβ and IFNλ1 production independent of IFN feedback loops in human T lymphocytes [68]. PRMT7 attenuates the binding of MAVS to TRIM31 and RIG-I by catalyzing the mono-methylation of MAVS at the R52 residue, but aggregated PRMT7 is incapacitated upon viral infection due to auto-methylation at the R32 residue, subsequently alleviating its suppressive effect on MAVS activation [69]. PRMT9 catalyzes the methylation of MAVS at the R41 and R43 residues, thereby inhibiting the aggregation and automatic activation of MAVS to maintain innate immune homeostasis [70] (Fig. 4).

### Acetylation in innate immunity

Protein acetylation, a key posttranslational modification controlled by acetyltransferases and deacetylases, plays functional roles in the innate immune response by adding acetyl groups to lysine residues in target proteins, thereby affecting the activity of signaling cascades [71].

Among all the acetylations of key proteins involved in innate immune signaling, the acetylation of RLRs has been extensively studied. Lysine (K) acetyltransferase 5 (KAT5) acetylates cGAS at several lysine residues in its N-terminal domain, promoting its ability to bind DNA and regulating the immune response to DNA viruses [72]. In addition, several cGAS acetylation sites (K7, K50, K384, K392, K394, and K414) have been discovered using mass spectrometry (MS). Among these residues, K384, K394, and K414 have been shown to inhibit cGAS function [73]. KAT8 directly interacts with IRF3 and mediates IRF3 acetylation at the K359 residue via its MYST domain, thereby reducing IRF3 transcriptional activity and suppressing antiviral innate immunity [74]. Beclin-1, an autophagy gene, interacts with the C-terminus of STING, causing K338 acetylation and autophagic degradation of STING [75]. Taken together, the acetylations on different lysine residues of the target protein by different acetyltransferases exhibit complex regulatory mechanisms.

Histone deacetylases (HDACs) are the most studied family of deacetylases of key signaling proteins [76]. TLR-inducible activation of HDAC7 enzymatic activity requires the MyD88 adaptor protein, except for the TLR3 agonist poly (I: C) [77]. On the other hand, MyD88 activity is directly regulated through lysine acetylation by HDAC6 [78]. HDAC3 directly deacetylates TBK1 at residues K241 and K692, leading to TBK1 activation and downstream IFN production [79]. HDAC6 deacetylates RIG-I and promotes RIG-I recognition and restriction of RNA virus infection [80]. Regarding NLRs, sirtuin 2 (SIRT2) deacetylates NLRP3, preventing age-related inflammation and insulin resistance, which can be reversed by NLRP3 K21/22/24R mutations [81]. SIRT3 interacts with and deacetylates NLRC4 at residues K71 or K272 to promote NLRC4 inflammasome activation, presumably to aid in the clearance of *S. Typhimurium* [82] (Fig. 4). Thus, as key modulators of innate immune responses, protein acetylation and deacetylation regulate the activation of substrates, resulting in downstream functional gene expression, limiting PAMP- or DAMP-induced inflammation, and maintaining immune homeostasis.

### Palmitoylation in innate immunity

Palmitoylation refers to the addition of palmitate to a cysteine residue, most commonly through a reversible thioester linkage (S-palmitoylation) [83]. Palmitoylation-induced protein-protein interactions, protein stability, and trafficking are critical for inflammation and innate immune responses [84].

Palmitoylation of membrane-bound TLR-2/5/10 and MyD88 is important for maintaining TLR signaling homeostasis [85]. Palmitoylation of TLR2, predominantly by zinc finger DHHC-type palmitoyltransferase 2/3/6/7/15 (ZDHHC2/3/6/7/15) at the C609 residue, is localized to a proximal transmembrane domain, which is important for TLR2-triggered signaling in dendritic cells [85]. TLR5 and TLR10 are potential palmitoylated proteins identified by chemoproteomics [85]. ZDHHC6 palmitoylates MyD88 at the C113 and C274 residues, which contributes to its interaction with IRAK4 [86]. Consistently, palmitoylation of membrane-bound TLRs mainly affects protein interactions and localization, which are critical for signaling.

In the nucleic acid-sensing system, palmitoylation of cGAS at the C474 residue is mainly catalyzed by ZDHHC18, which inhibits cGAS dimerization in human and mouse cell lines [87]. The palmitoylation of STING at the C88/91 residues by ZDHHC3/7/15 is essential for its assembly into multimeric complexes at the Golgi apparatus, thus allowing it to recruit both TBK1 and IRF3 [88, 89]. STING activation in platelets is a critical driver of sepsis-induced pathology. Sepsis-derived cGAMP promoted STING binding to STXBP2, SNARE complex assembly, granule secretion and subsequent septic thrombosis, which is likely dependent on STING palmitoylation [90]. The mitochondrial protein CPT1A recruits the endoplasmic reticulum-localized ZDHHC4 to catalyze MAVS C79 palmitoylation, which increases MAVS stability and activation by inhibiting K48-linked ubiquitination but facilitating K63-linked ubiquitination, thereby enhancing the IFN-I response and enhancing control of viral infection [91].

**Fig. 5:**
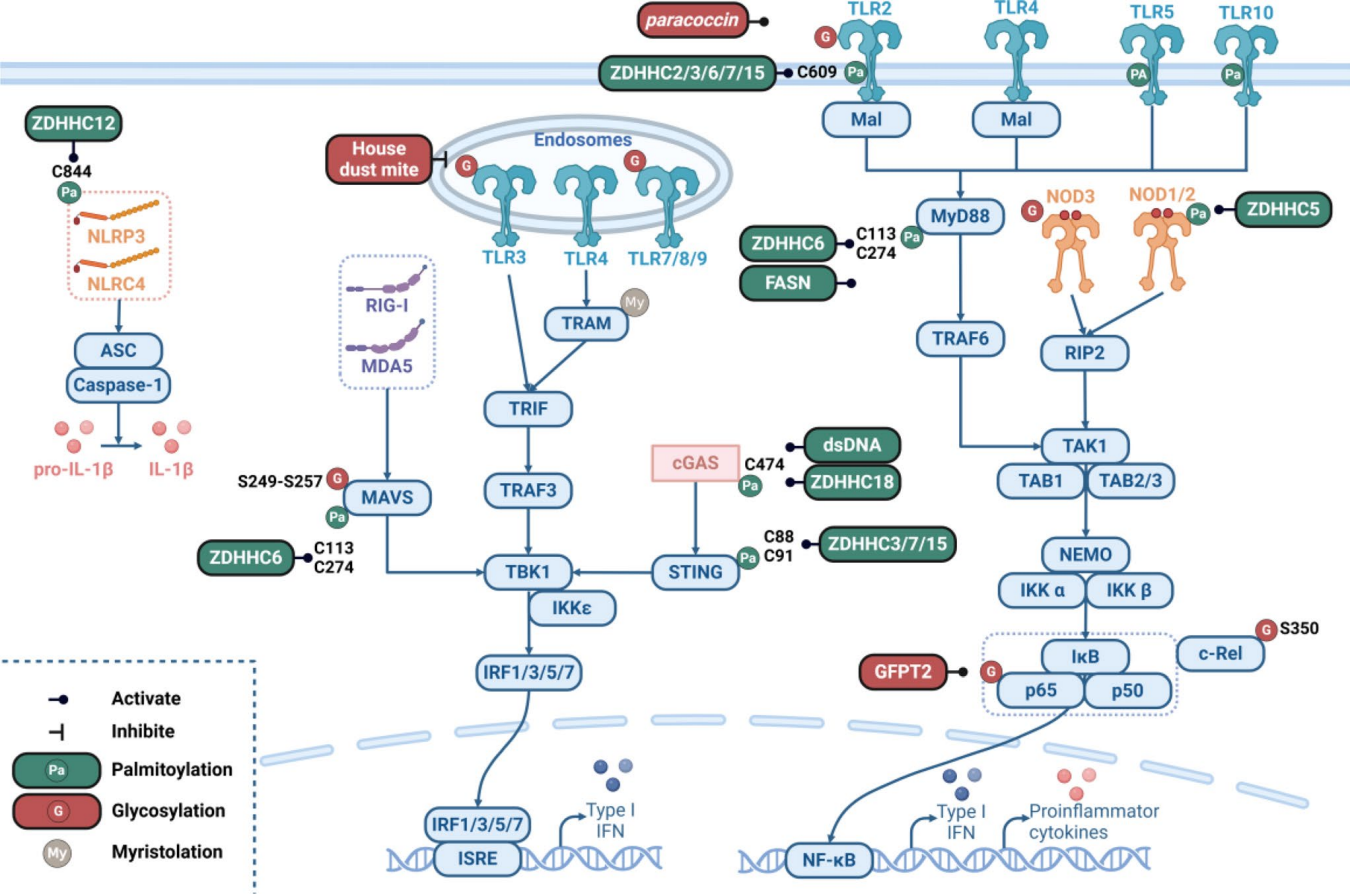
Regulation of PRR signaling pathways by other unconventional PTMs. Other unconventional PTMs, including palmitoylation, glycosylation, and myristoylation, often modify cysteine (C), serine (S), asparagine (N), and lysine (K). The color differences indicate the various strategies used for targeting the proteins. The blue boxes represent key molecules in PRR signaling pathways. A line with a dot indicates positive regulation, a line with a vertical bar indicates negative regulation, and the modification site is labeled next to it

With respect to NLRs, S-palmitoylation acts as an indispensable element to stabilize NOD1/2 and a brake to turn off the NLRP3 inflammasome. ZDHHC5-mediated NOD1/2 S-palmitoylation at a cysteine thiol is required for membrane localization and stability [92]. Defective S-palmitoylation of NOD1/2 leads to severe immunologic and inflammatory diseases, including Crohn’s disease (CD), ulcerative colitis (UC), Blau syndrome, Behcet’s syndrome, early-onset sarcoidosis (EOS), and atopic diseases [92–94]. ZDHHC12-mediated palmitoylation of NLRP3 at the C844 residue enhances its recognition by heat shock protein family A (Hsp70) member 8 (HSPA8/HSC70) and degradation through the chaperone-mediated autophagy pathway [95, 96]. Defective palmitoylation is implicated in several disease-associated mutations (21 H, S102L, R490K, or G571R) of NLRP3, increasing NLRP3 protein stability and contributing to overt NLRP3 inflammasome mobilization [95] (Fig. 5). Unlike TLRs, which are located on the cell membrane, the key proteins of RLRs and NLRs are mostly located in the cytoplasm. Thus, in addition to affecting membrane localization, palmitoylation of these proteins affects intracellular protein aggregation, trafficking, and protein stability, which are essential for signaling.

### Glycosylation in innate immunity

Glycosylation, the covalent attachment of glycoconjugates to target proteins or lipids, plays a critical role in cellular adhesion to the extracellular matrix and protein-ligand interactions within cells [97].

In TLR signaling, the extracellular domains (ECDs) of human and mouse TLR2 have four (N114, N199, N414, and N442) and three (N147, N414, and N442) N-glycosylation sites, respectively [98]. Glycosylation occurs on the inner and outer surfaces but not on the lateral surface where ssRNA binds to TLR7 [99]. Various pathogen lectins directly target TLR N-glycans through carbohydrate recognition domains [100]. For example, paracoccin (PCN), an N-acetylglucosamine-binding lectin from the human pathogenic fungus *Paracoccidioides brasiliensis*, attaches to the fourth N-glycan of the TLR2 ectodomain and initiates the Th1 immune response [101]. Furthermore, the key downstream signaling proteins of TLRs can be glycosylated. NF-κB subunit C-Rel possesses O-GlcNAcylation on the serine 350 (S350) residue upon C-Rel binding to DNA or transactivation [102]. Glutamine fructose-6-phosphate amidotransferase 2 (GFPT2) enhances the O-glycosylation of the p65 subunit NF-κB, leading to the nuclear translocation of p65 and NF-κB pathway activation [103, 104]. In RLR pathways, MAVS contains a profusely O-GlcNAcylated serine-rich region between sites 249 and 257, the modification of which hinders the interaction of MAVS with TRAF3, subsequently preventing IRF3 activation and IFNβ production [105] (Fig. 5). As N-glycosylation is targeted to asparagine and O-glycosylation is targeted to serine, threonine, and hydroxylysine, N-glycosylation of TLRs affects the stability of proteins, while O-glycosylation of TLRs and RLRs participates in signal transduction and other processes of proteins, including interference with protein phosphorylation and nuclear translocation.

### Myristoylation in innate immunity

Myristoylation is the modification of a protein with myristate, a hydrophobic 14-carbon fatty acid. Myristoylation typically affects the subcellular trafficking, localization and stability of cytoplasmic eukaryotic proteins [106] and is critical for regulating protein physiological functions and immune responses [107]. Myristoylated TIR domain-containing adaptor molecule (TRAM) colocalizes with TLR4 at the plasma membrane and in the Golgi apparatus. However, the myristoylation mutants (Met-Gly-Xaa-Xaa-Xaa-Ser and Lys) of TRAM-G2A cannot efficiently signal membrane localization dysfunction [108]. As a cellular myristate binding protein, heme oxygenase 2 (HO-2) binds to TRAM and inhibits the LPS-TLR4 signaling pathway [109] (Fig. 5). To date, research progress on the effect of myristoylation on the innate immune response has been limited to the TLR pathway, and further investigations of other innate immune signaling pathways are required in the future.

## Discussion

Recent research on unconventional PTMs in the context of innate immunity has made significant advances, shedding light on the intricate regulatory mechanisms that govern immune responses. Although conventional PTMs, such as phosphorylation and ubiquitination, have been extensively studied, the emerging focus on unconventional PTMs, including unanchored ubiquitination, ISGylation, SUMOylation, NEDDylation, methylation, acetylation, palmitoylation, glycosylation, and myristylation, represents a new frontier. Compared to research on conventional PTMs, research on unconventional PTMs is still in its infancy and faces the challenge of revealing the specific roles of each modification and their interconnectivity within immune signaling cascades. Future investigations should focus on unravelling the precise molecular mechanisms underlying each unconventional PTM and their crosstalk with conventional PTMs.

Multiple proteins are affected by more than one kind of PTM. The positive and negative regulatory interplay between multiple PTMs in a protein contributes to a dynamic equilibrium, increasing the functional diversity and flexibility to respond to manifold scenarios. For example, SENP2 negatively regulates the SUMOylation of RIG-I and MDA5 at the same residues, modulating efficient innate immunity to RNA viruses and their timely termination [37]. PTM crosstalk preferentially occurs in key protein domains, such as RNA recognition motifs, protein kinases, and transcription factors, which participate in various biological processes, including RNA processing, DNA damage response, signal transduction, and cell cycle regulation. One protein could play different PTM roles in the innate immune response. Each modification can lead to distinct changes in protein activity or interactions with other molecules, allowing them to participate in various aspects of the immune response. TRIM28 possesses E3-SUMO ligase activity, catalyzing the SUMOylation of key signaling proteins. As mentioned, TRIM28 can SUMOylate the NLRP3 protein, maintaining its stability and promoting downstream immune-inflammatory responses [46]. Additionally, TRIM28 also exhibits ubiquitination activity. Through K48-linked polyubiquitination, TRIM28 transfers ubiquitin chains to residues K7, K10, K371, K420, and K500 of MAVS, targeting MAVS for ubiquitin modification, thus negatively regulating the RLR signaling pathway and suppressing the production of downstream type I interferons and proinflammatory cytokines [110]. SUMOylation and ubiquitination are two distinct forms of posttranslational modifications (PTMs), suggesting that a gene/protein can undergo different types of PTMs. However, despite their differences, SUMOylation and ubiquitination share certain similarities in terms of their mechanisms and principles, such as the involvement of similar enzyme types, such as the ubiquitin-activating enzyme E1, ubiquitin-conjugating enzyme E2, and ubiquitin ligase E3. Thus, caution should be taken when developing therapies targeting one gene/protein that may be involved in multiple types of PTMs. Whether more genes/proteins can undergo multiple vastly different PTMs remains to be further investigated.

In addition, elucidating the functional consequences of dysregulated unconventional PTMs in disease pathogenesis will be crucial for identifying potential therapeutic targets and advancing our understanding of immune-related disorders, such as cancers, neurodegeneration, and infections. An increase in treatments targeting enzymes or key proteins involved in PTM-associated modification has been demonstrated. For instance, the NF-κB inhibitor JSH-23 mimicked the effects of neddylation inhibition in the early phase of HSV-1 infection [111]. Aspirin acetylates cGAS at the K384, K394, or K414 residue to inhibit cGAS-mediated immune responses [73]. Covalent small-molecule inhibitors (C-178, C-176, or 2-bromopalmitate) block the activation-induced palmitoylation of STING, reducing STING-mediated inflammatory cytokine production [112]. However, drug design targeting other unconventional PTMs, such as SUMOylation, methylation, and myristoylation, requires more effort. As the field progresses, the integration of unconventional PTMs into the broader landscape of innate immunity will likely unveil novel avenues for therapeutic interventions and diagnostic strategies.

In summary, unconventional PTMs play a pivotal role in modulating innate immune signaling pathways, increasing the complexity of the regulatory network. Unlike their conventional counterparts, unconventional PTMs exhibit unique regulatory functions that contribute to the precise control of immune sensors, signaling protein structure, stability, activity, localization, and interactions with other biomolecules. Understanding the nuances of these unconventional PTMs provides a more comprehensive view of immune homeostasis and its dysregulation in various disorders.

## Data Availability

Not applicable.
